# Different Calculation Strategies Are Congruent in Determining Chemotherapy Resistance of Brain Tumors In Vitro

**DOI:** 10.3390/cells9122689

**Published:** 2020-12-15

**Authors:** Igor Fischer, Ann-Christin Nickel, Nan Qin, Kübra Taban, David Pauck, Hans-Jakob Steiger, Marcel Kamp, Sajjad Muhammad, Daniel Hänggi, Ellen Fritsche, Marc Remke, Ulf Dietrich Kahlert

**Affiliations:** 1 Clinic for Neurosurgery, Medical Faculty, Heinrich-Heine University Düsseldorf, 40225 Düsseldorf, Germany; Ann-Christin.Nickel@med.uni-duesseldorf.de (A.-C.N.); Hans-Jakob.Steiger@med.uni-duesseldorf.de (H.-J.S.); MarcelAlexander.Kamp@med.uni-duesseldorf.de (M.K.); Sajjad.Muhammad@med.uni-duesseldorf.de (S.M.); Daniel.Haenggi@med.uni-duesseldorf.de (D.H.); 2 Department of Pediatric Oncology, Hematology, and Clinical Immunology, Medical Faculty, University Hospital Düsseldorf, 40225 Düsseldorf, Germany; Nan.Qin@med.uni-duesseldorf.de (N.Q.); Kuebra.Taban@med.uni-duesseldorf.de (K.T.); David.Pauck@med.uni-duesseldorf.de (D.P.); Marc.Remke@med.uni-duesseldorf.de (M.R.); 3 Department of Pediatric Neuro-Oncogenomics, German Cancer Consortium (DKTK), 69120 Heidelberg, Germany; 4 German Cancer Research Center (DKFZ), 69120 Heidelberg, Germany; 5 IUF—Leibniz Research Institute for Environmental Medicine, 40225 Düsseldorf, Germany; Ellen.Fritsche@IUF-Duesseldorf.de; 6 Medical Faculty, Heinrich-Heine-University, 40225 Düsseldorf, Germany; 7 Beijing Neurosurgical Institute, Capital Medical University, Beijing 100050, China

**Keywords:** glioblastoma, in vitro pharmacology, quantification, drug response, mathematical modeling, off-target risk

## Abstract

In cancer pharmacology, a drug candidate’s therapeutic potential is typically expressed as its ability to suppress cell growth. Different methods in assessing the cell phenotype and calculating the drug effect have been established. However, inconsistencies in drug response outcomes have been reported, and it is still unclear whether and to what extent the choice of data post-processing methods is responsible for that. Studies that systematically examine these questions are rare. Here, we compare three established calculation methods on a collection of nine in vitro models of glioblastoma, exposed to a library of 231 clinical drugs. The therapeutic potential of the drugs is determined on the growth curves, using growth inhibition 50% (GI${}_{50}$) and point-of-departure (PoD) as the criteria. An effect is detected on 36% of the drugs when relying on GI${}_{50}$ and on 27% when using PoD. For the area under the curve (AUC), a threshold of 9.5 or 10 could be set to discriminate between the drugs with and without an effect. GI${}_{50}$, PoD, and AUC are highly correlated. The ranking of substances by different criteria varies somewhat, but the group of the top 20 substances according to one criterion typically includes 17–19 top candidates according to another. In addition to generating preclinical values with high clinical potential, we present off-target appreciation of top substance predictions by interrogating the drug response data of non-cancer cells in our calculation technology.

## 1. Introduction

Performing cell growth analysis in vitro using patient-derived disease models is the gold standard for various academic and industry-driven cancer research projects. Historically, this approach has led to the identification of most of the current block buster anticancer drugs [1]. Although successful, with the recent rapid technological advances, inconsistencies when trying to replicate in vitro cancer pharmacology approaches have been identified [2,3] with both technical [4] and biological [5] factors being identified to contribute to the reproducibility hurdles.

Chemotherapy effects on cancer cells are measured using various methods depending on the biological question [6,7]. In modern throughput campaigns, the use of bioenergetic quantification [8] is established as a reliable sensitive method to report the anti-cell growth potential of drugs. Multiple methods of expressing the effectiveness of a drug in in vitro experiments are common in practice. Assuming a drug shows an effect, its potency will depend on the drug concentration. The dependency between the concentration and the effect is commonly modeled by the Hill equation [9,10,11]. In its simplest form, when it is used to model cell viability in a drug solution and when the viability is known to span the range 0–1 (in percentage: 0–100%), it is a logistic function over the logarithm of the drug concentration and is fully determined by two parameters, the slope and the x-offset. When the y-range of the curve is different, e.g., because it is used to model the absolute number of surviving cells (or its correlate, but in any case, a value that can be much larger than one) and never falls to zero (because a fraction of cells survive even in the saturated solution), two additional parameters are required. They determine the upper and the lower bound of the curve. There are multiple, equivalent ways of writing this four-parameter equation. In this study, we use the following form, where its relationship with the common logistic function is more apparent and whose parameters are readily interpretable:

(1) $$n\left(\mathrm{logC}\right)=A_{0}\cdot \left(b_{\infty }+\frac{1-b_{\infty }}{1+e^{-\beta \cdot (\mathrm{logC}-{\mathrm{logIC}}_{50})}}\right)$$

**Figure 1:**
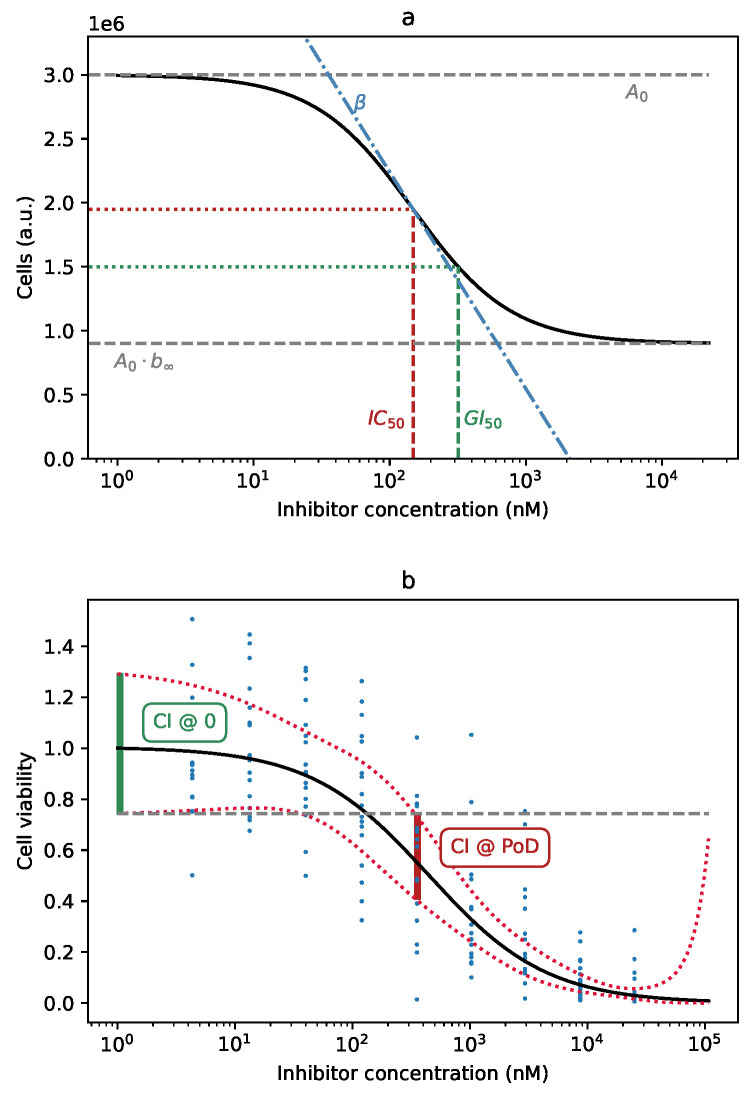
(**a**) Graphical representation of the parameters of the Hill equation: $A_{0}$: the curve upper bound; $b_{\infty }$: the curve lower bound, as a fraction of $A_{0}$; ${\mathrm{logIC}}_{50}$: the logarithm of the substance concentration at the inflection point of the curve, $IC_{50}$; β: the parameter controlling the slope of the curve. Note that GI${}_{50}$ is not itself a parameter of the curve; it is the point at which the curve falls to 50% of its maximum value, $A_{0}$. (**b**) Definition of the point-of-departure (PoD), based on the confidence band of the curve. The existence of a drug effect can be established with 95% confidence at the lowest concentration at which the Hill curve’s confidence interval (CI, red) does not overlap with the 95% CI at logC=0 (green).

Here, n(logC) is the total number of cells (or its correlate, like the measured intensity of a fluorescent marker) for the specific drug concentration *C*, with logC=log(C). $A_{0}$ is the upper bound of the curve, the number of surviving cells in the absence of the drug. $b_{\infty }$ is the lower bound of the curve, as a fraction of the total. It is normally a value between zero and one, but can be greater than one if the drug actually stimulates cell growth. The parameter β is the usual slope parameter of the logistic curve and ${\mathrm{logIC}}_{50}$ the logarithm of IC${}_{50}$, the drug concentration at which the curve falls halfway between its upper and lower bound [12]. It is the inflection point of the curve, the point at which the curve is at its steepest (Figure 1a). Fitting the Hill curve to the empirical cell survival data amounts to finding the appropriate set of parameters.

The first parameter, $A_{0}$, depends on the starting number of cells, the incubation time, and the method used for their counting. It describes the experiment setup and is not related to the tested substance. The remaining three parameters are indicative of the drug effectiveness. $b_{\infty }$ tells us what is the maximum possible effect that can be achieved by the drug. ${\mathrm{logIC}}_{50}$ is the break-even point, where the drug reaches half of its potential, and β describes how rapidly the effect increases with the increasing drug concentration.

To summarize drug effectiveness in a single number and make it easier for comparison, researchers have come up with derived measures. One common measure is GI${}_{50}$, a paramter defined by the National Cancer Institute COMPAREmethod initiative [13]. It is the concentration at which the cell growth is inhibited by 50%. When $b_{\infty }=0$, GI${}_{50}$ = IC${}_{50}$, but, in general, GI${}_{50}$ is larger. It is easy to confuse GI${}_{50}$ and IC${}_{50}$ (or EC${}_{50}$, which is used as a synonym [12]). IC${}_{50}$ or EC${}_{50}$ is just a parameter for mathematically defining the Hill curve, but it has no direct interpretation in terms of cell growth. In [14], the authors explicitly warn: “Don’t overinterpret the EC50. It is simply the concentration of agonist required to provoke a response halfway between the baseline and maximum responses [...] it is not a direct measure of drug affinity.” To add to the confusion, some sources, like https://www.graphpad.com/support/faq/relative-vs-absolute-ic50/, use “relative IC${}_{50}$” to mean IC${}_{50}$ and “absolute IC${}_{50}$” to refer to GI${}_{50}$.

Another measure is the area under the curve (AUC). It is the integral of the four-parameter Hill equation and can be computed analytically. In practice, however, the computation involves some decisions by the researcher. The integral under the whole Hill curve is infinite, because the curve has a finite, non-zero value, over an infinite range of log-concentrations. In order to get finite AUC values, the integration has to be limited to some reasonable range, e.g., one order of magnitude below and above the lowest and highest drug concentrations, respectively.

Yet another possible effectiveness measure is the point-of-departure (PoD). The measure is common in in vivo trials and describes the lowest dosage at which an effect can be observed. Again, in order to apply it to the Hill curve, some decisions have to be made. As the Hill curve is smooth, there is always some effect; it can be minuscule, but it is never exactly zero. It is simple to define PoD as the concentration at which the viability falls to 95%, or 90%, or some other percentage. If we settle for 50% viability, PoD becomes identical to GI${}_{50}$. Another possibility is to define PoD as the concentration at which the effect can be observed with some pre-defined confidence, say 95%. As the parameters of the Hill curve are determined from noisy, empirical data, the shape of the curve is uncertain to a degree. This level of uncertainty is graphically shown as the confidence band around the curve. It can be interpreted as an infinite collection of point-wise confidence intervals (CI) around the curve, for every possible drug concentration. The point-of-departure in the above sense is the lowest concentration for which the CI does not overlap with the CI at zero log-concentration (Figure 1b). In this study, we use PoD with this latter meaning.

Whether and how the choice of effectiveness measure influences research results are still not fully understood. In order to shed some light on this, we tested a large number of substances for their effectiveness on different cell lines and compared their rankings according to different measures.

3D in vitro systems of cancer are the current state-of-the-art to model the disease as they more closely recapitulate the stem cell properties of malignant cancers as compared to classical monolayer cultures [15,16,17]. 2D assays are the basis of most of the large consortial cancer in vitro pharmacology projects [18,19]. For the present study, we used stem cell models of glioblastoma, the most commonly occurring brain-born cancer. Standard clinical treatment currently includes a combination of chemotherapy and radiotherapy, but the prognosis is still poor, with a median overall survival of only 14.6 months [20].

The implementation of automated processes in work procedures is a standard dogma in industry and in clinical labs, with the aim of achieving high accuracy, transparency, and effectivity. The development of low-cost lab automation led to a wider distribution of printing or robotic devices for substance testing projects in academic labs with limited resources [21], increasing the value of preclinical deliverables [22]. Our project applied a wide collection of 3D in vitro systems of brain cancer and exposed them to a collection of clinically approved drugs using an automation device. Anti-growth effects were reported using the metabolic activity measurement CellTiterGlow^®^ assay. The presented results are an interdisciplinary compendium of state-of-the-art disease modeling, state-of-the-art laboratory procedures, and innovative statistical modeling to investigate a biotechnological and socio-economical relevant challenge.

The study was conducted in accordance with the Declaration of Helsinki, and the protocol was approved by the Ethics Committee of the Medical Faculty of the Heinrich-Heine University (Study ID #5206).

## 2. Materials and Methods

### 2.1. Mathematical Background

Finding the parameters for which the Hill curve closely fits the empirical values is commonly done by a least-squares method, which minimizes the sum of the squared differences between the data and the curve. Least-squares imply a Gaussian distribution of the residuals, with a constant variance. This assumption is increasingly violated as survival values approach zero. Since survival can never be negative, the actual distribution of the residuals becomes increasingly asymmetric, right-skewed. A theoretically founded way of coping with this would require a probabilistic model for the errors, which can be quite complicated in practice. There are many factors contributing to the error, from the precision in the drug dosage, to the homogeneity of the cell suspension, to the accuracy of fluorescence measurement, to name just a few.

A heuristic alternative would be to perform the fitting on logarithmically transformed data, with the corresponding logarithmic transformation of the Hill curve (the logarithm is a special case of the more general Box–Cox transform, commonly used in statistics). The decreasing tail of the Hill curve closely approximates a falling exponential function as the log-concentration goes towards infinity, so taking the logarithm transforms it into a straight line. Least-squares fitting on such transformed data and in that value range is basically a linear regression.

**Figure 2:**
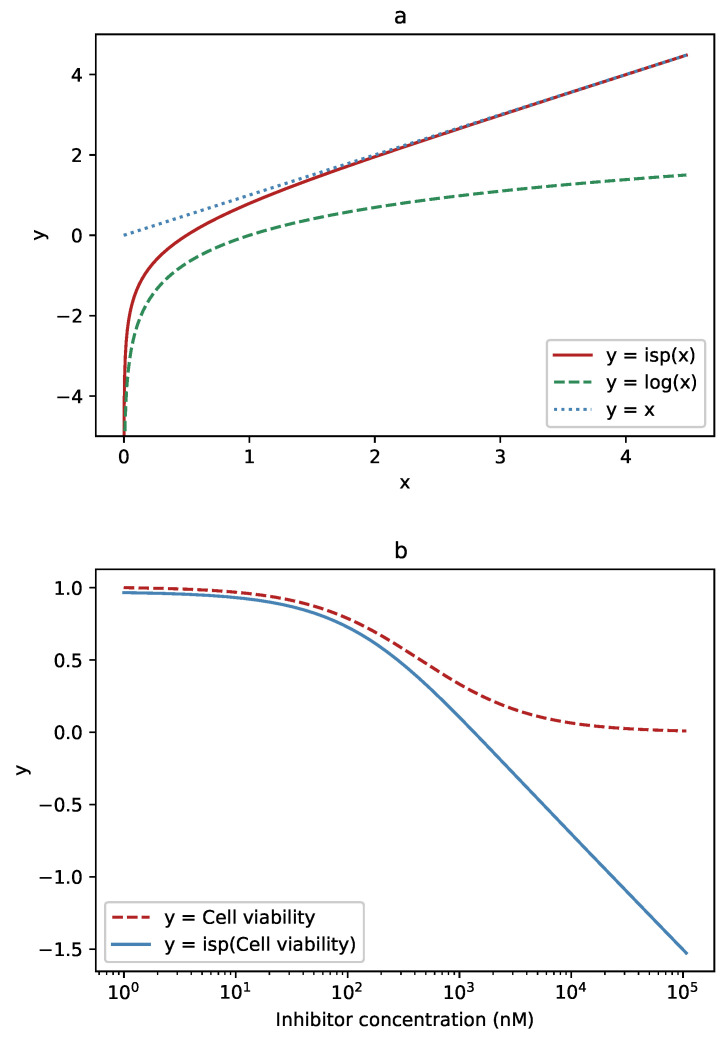
The function used to transform the data and its effect on the Hill curve: (**a**) For low values, close to zero, the inverse softplus function (solid, red) approximates the logarithm (dashed, green). For high values, it approaches the identity line, y=x (dotted, blue). (**b**) Where the logistic (Hill) function (dashed, red) has high values, the inverse softplus (solid, blue) leaves it almost unchanged. At low values, where the logistic function becomes close to a falling exponential, the inverse softplus transforms it to an almost straight line.

However, this approximation is not valid for large values, near the top, flat part of the Hill curve. In that range, we would prefer to perform the fitting on the original, untransformed data. To have the best of both worlds, the inverse softplus transform can be used. The inverse softplus function,

(2) $$\mathrm{isp}\left(x\right)=s\cdot \ln\left(e^{x/s}-1\right)$$

has the convenient property of being close to linear for large *x* and close to logarithmic near x=0 (Figure 2a). Applied to the Hill curve, it leaves large values mostly unchanged, but straightens its exponentially decreasing tail towards a straight line (Figure 2b). The transition between the two is smooth, and the dominant behavior at the value *x* depends on the scaling factor *s*.

### 2.2. Cell Models and Experimental Setup

We used a collection of 9 stem cell models of glioblastoma (HSR-GBM1 [23]; JHH520 [24]; NCH421k, NCH644 [25,26]; BTSC-23, BTSC-233, BTSC-268, BTSC-349, BTSC-407 [27]), and exposed them to a library of clinical drugs (231 substances, each in 9 different concentrations; 4.33 nM to 25 μM) using a semi-automated screening platform. For each cell model, repetitive drug resistance tests were performed: two biological (except for BTSC-23) and three technical replications. For each biological replication, the mean of the technical replications was used. Undifferentiated and immortalized human neural progenitor cells (ReNcell^®^CX, Sigma-Aldrich, St Louis, MO, USA) and neural stem cells (H9-Derived, Gibco) were used as healthy controls to evaluate the toxicity of the drugs. Additionally, we also tested the inhibition efficiency using three different normal adult human dermal fibroblasts (NHDF-Ad, Lonza, Basel, Switzerland). Effects on cell growth were assessed 72 h after substance exposure using the CellTiterGlow^®^ assay (Promega, Madison, WI, USA). All procedures were in consent with the local ethical commission oversights.

### 2.3. Fitting the Curves

**Figure 3:**
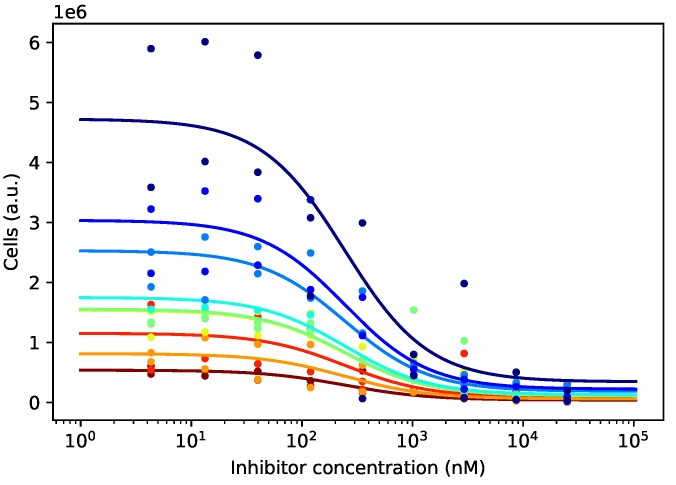
Different cell lines have different growth patterns, and after 72 h of incubation, their numbers differ significantly at every drug concentration. Curves differing only in the amplitude parameter $A_{0}$ and sharing the remaining three parameters, $b_{\infty }$, logC, and β, were fitted to the empirical data. The different amplitudes were later used for normalizing the curves.

For each candidate substance, we fitted the four-parameter Hill curve. Since the starting cell concentrations differed for different cell lines, we first needed to normalize the empirical cell numbers so that the corresponding curves started from the same reference point. To that end, we fitted a curve for each cell line, having its individual amplitude $A_{0}$, but sharing the remaining three parameters with the other cell lines. This ensured that the curves shared an identical shape, irrespective of the cell line, and differed only in their height (Figure 3). The fitting was performed by the ordinary least-squares procedure on the raw data.

We then normalized the empirical cell counts by dividing them by the modeled cell count at zero log-concentration. This produced scaled cell counts, all starting around 1 at the lowest substance concentration and behaving similarly as it increased.

We re-fitted a single four-parameter Hill curve through the normalized data. In order to ensure a physically plausible, non-negative confidence band, we applied the least-squares procedure on the data further transformed by the inverse softplus function. We chose the scaling factor s so that for x>0.5, the behavior is closer to linear and closer to logarithmic for x<0.5. In other words, cell viabilities above 50% were modeled by a function approximating the Hill equation itself and the viabilities below 50% by a function approximating its logarithm. The confidence bands were computed using the standard delta method on the transformed curves and transformed back using the softplus function:

(3) $$\mathrm{sp}\left(x\right)=s\cdot \ln\left(e^{x/s}+1\right)$$

We relied on the computed 95% confidence bands to determine the point-of-departure (PoD), as described in the Introduction.

All computations were performed in Python, Version 3.7.7, using the NumPy and SciPy libraries and our own code.

### 2.4. Quantifying the Drug Effect

The therapeutic potential of the drugs was evaluated on the fitted viability curves using GI${}_{50}$, GI${}_{90}$, GI${}_{95}$, AUC, and PoD. If GI${}_{50}$, GI${}_{90}$, GI${}_{95}$, or PoD was not reached within the tested concentrations, it was assigned the value “infinite”. To determine whether a drug had an effect, we relied on GI${}_{50}$ and, independently, PoD having finite values. If the two criteria were not in agreement, we considered both possibilities, according to each of them. As the AUC always has a finite value, it alone cannot be used to determine whether a drug has an effect. However, in conjunction with GI${}_{50}$ or PoD, a threshold could be established.

**Figure 4:**
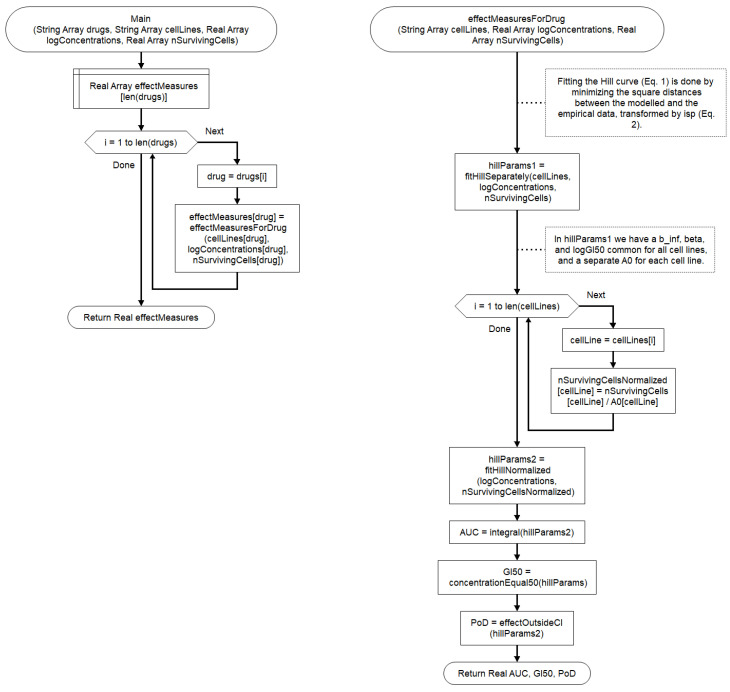
The workflow for calculating the effect measures for all drugs and cell lines.

The high-level algorithm used for the computation is shown in Figure 4.

## 3. Results

Using GI${}_{50}$ as the criterion, an effect was observed on 83 substances (36%). When PoD was used as the criterion, an effect was observed on 63 substances (27%). GI${}_{90}$ and GI${}_{95}$ were much more lenient, with GI${}_{90}$ claiming an effect on 150 substances (65%) and GI${}_{95}$ even on 166 drugs (72%).

**Figure 5:**
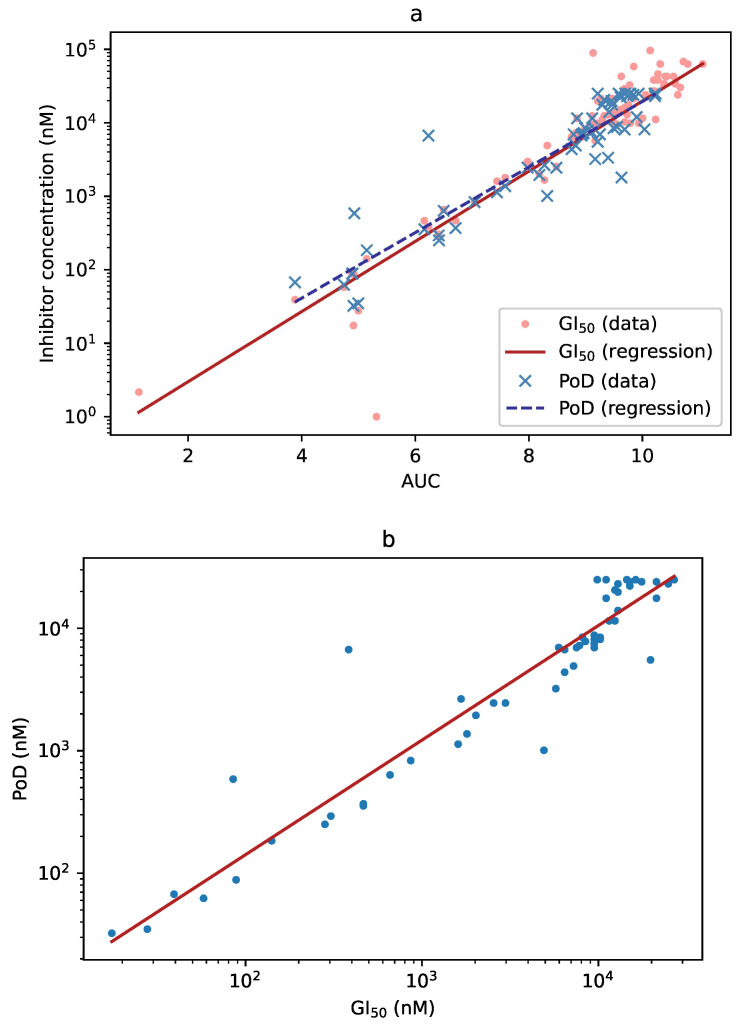
(**a**) Correlation between the AUC and GI${}_{50}$ (dots, solid line) and between the AUC and PoD (crosses, dashed line). The three measures can be used more-or-less interchangeably for detecting substance effect. (**b**) Correlation between GI${}_{50}$ and PoD. When both values could be computed (i.e., neither was infinite), they were very similar. Note, however, that PoD also depends on the experimental setup (see Discussion below).

When an effect was detected using GI${}_{50}$ as the criterion, the GI${}_{50}$ value and AUC were highly correlated ($R^{2}=0.836$, $p<10^{-23}$). Furthermore, when an effect was detected using PoD, the PoD and the AUC values were as well highly correlated ($R^{2}=0.927$, $p<10^{-26}$; Figure 5a). Finally, when both GI${}_{50}$ and PoD detected an effect, their respective values were highly correlated, as well ($R^{2}=0.949$, $p<10^{-29}$; Figure 5b).

**Figure 6:**
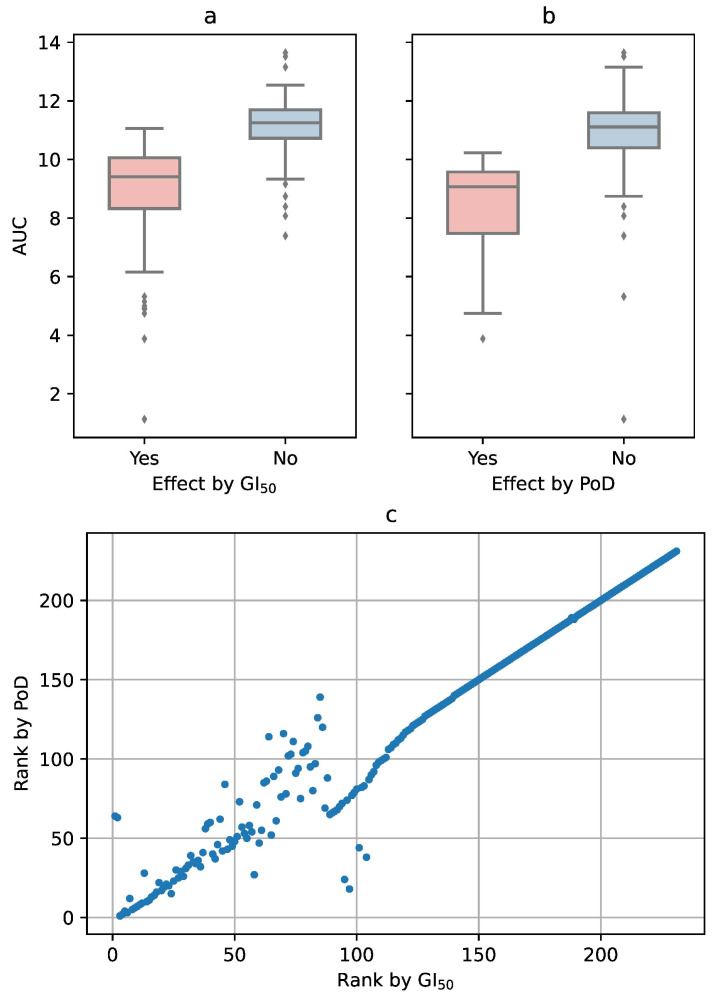
(**a**) If a drug failed to reach GI${}_{50}$, the AUC had a significantly higher value. (**b**) The same behavior, only with slightly lower AUC values, was observed when using PoD as the criterion for the effect. (**c**) Correlation between the rankings by GI${}_{50}$ and by PoD. For the top 20 substances (lower left corner in the figure), there is little difference between the two criteria.

Using GI${}_{50}$ as the criterion, AUC had a significantly higher value (t-test, $p<10^{-27}$) when no effect could be observed than when GI${}_{50}$ had a finite value (mean =11.15 vs. mean =8.77; Figure 6a). When PoD was taken as the criterion, the result was similar, only with slightly shifted AUC values (mean =10.92 vs. mean =8.34, $p<10^{-26}$; Figure 6b). This suggests that a threshold on AUC can be defined (for our set of drugs and tested concentrations: between 9.5 and 10), so that the AUC with a value above it would also indicate the lack of an effect of the tested drug.

**Figure 7:**
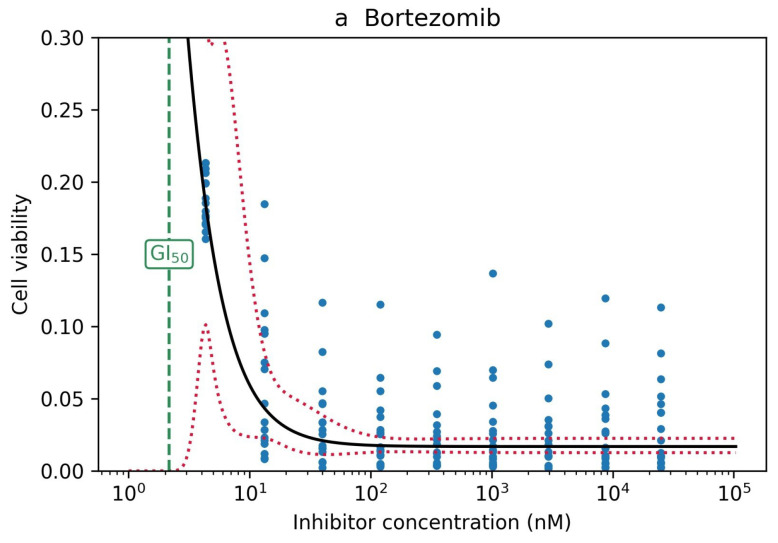

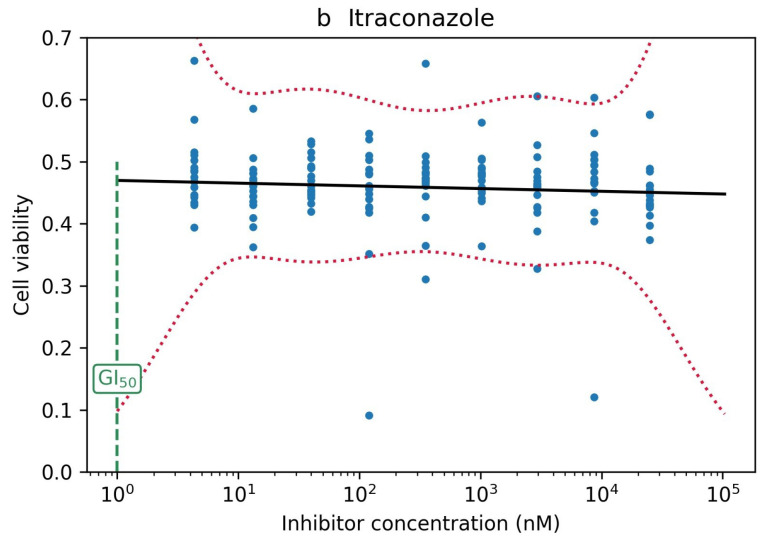
(**a**) Bortezomib showed an effect very early, at concentrations for which there were no measurements, so the confidence band was very wide. (**b**) Itraconazole did not have an effect, and the numeric algorithm failed to fit a logistic curve to the empirical data.

Ranking of the drugs by their effectiveness was not identical, but highly similar for GI${}_{50}$ and PoD as criteria, at least for the top-ranking substances (Figure 6c). Among the top 20 drugs ranked by GI${}_{50}$ were also 16 of the top 20 drugs ranked by PoD and 19 of the top 20 drugs ranked by the AUC (Table 1). Interestingly, two drugs ranked 1 and 2 by GI${}_{50}$, itraconazole and bortezomib, never reached a PoD. For bortezomib, it is obviously a regression issue: The drug is so effective that it causes low cell viability even at low concentrations, for which we did not perform any measurements. Consequently, the confidence interval at such low concentrations turned out to be very wide (Figure 7a). For itraconazole, the issue was the opposite: the drug is obviously ineffective (Figure 7b), and the numerical fitting algorithm did not converge to a reasonable set of parameters.

**Figure 8:**
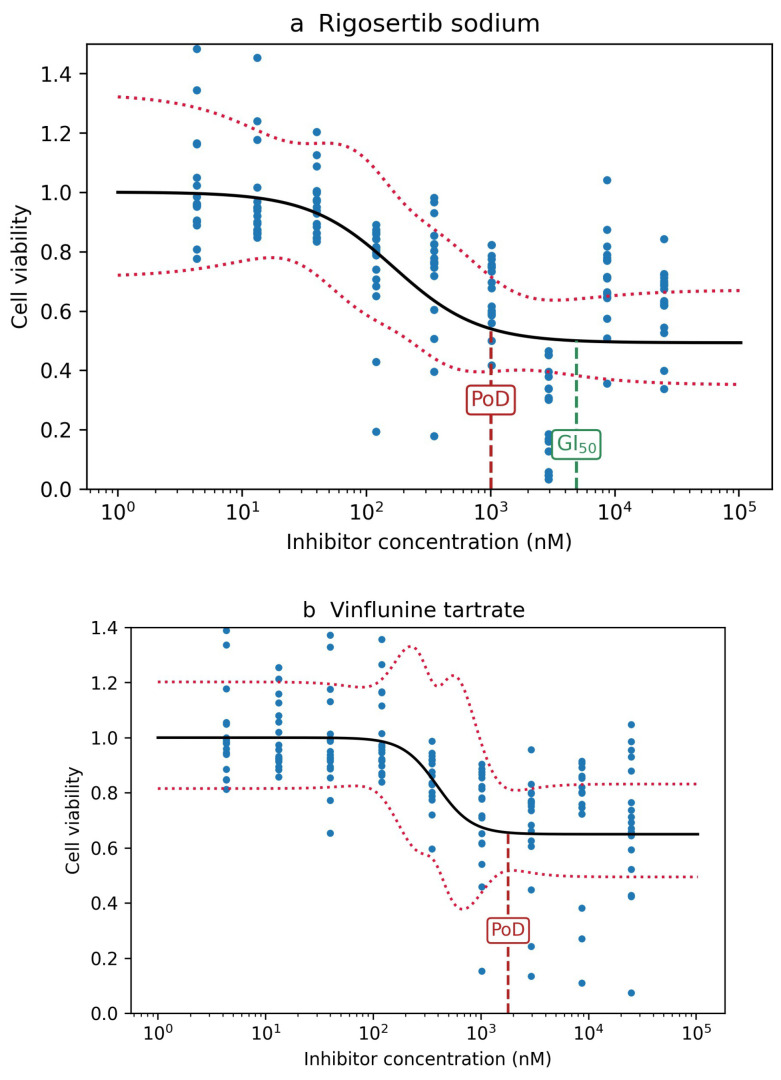
(**a**) Rigosertib sodium leveled off shortly after reaching GI${}_{50}$. (**b**) Vinflunine tartrate leveled off before reaching GI${}_{50}$.

Conversely, among the top 20 drugs ranked by PoD were 16 of the top 20 drugs ranked by GI${}_{50}$ and 16 of the top 20 drugs ranked by AUC (Table 2). Here, no effect was detected for vinflunine tartrate (Rank 18) when using GI${}_{50}$ as the criterion. The drugs actually showed an effect at low concentrations, but it leveled off above 50% viability, so that GI${}_{50}$ was never reached. Rigosertib sodium (Rank 15 by PoD and 24 by GI${}_{50}$) showed a similar behavior, but leveled off shortly after falling below 50% (Figure 8).

Finally, the top 20 drugs ranked according to the AUC included 19 of the top 20 substances ranked by GI${}_{50}$ and 16 of the top 20 ranked by PoD. For GI${}_{50}$, only fludarabine phosphate (which had an GI${}_{50}$ value and a PoD and, consequently, should be considered effective) was substituted by vincristine sulfate, which is actually ineffective. In addition, in the PoD ranking, ponatinib (Ranked 21 by the AUC), doxorubicin hydrochloride (23), rigosertib sodium (25), and the above-mentioned vinflunine tartrate (63) were missing.

For practical purposes, the effect on cancer cells is not the only criterion for selecting substances. At least as important is having low unwanted toxic effects on cells. We used the above-described method to model the drug effect on five healthy cell cultures and calculated the drug toxicity as $1-f_{s}$, $f_{s}$ being the fraction of the surviving cells. The toxicity effects on healthy cells at the GI${}_{50}$ and PoD concentrations are also given in Table 1 and Table 2. The data for five substances (ixazomib (MLN9708), copanlisib (BAY 80-6946), sapanisertib (INK 128), auranofin, and tanespimycin (17-AAG)) were missing due to an infrastructure reason, because the healthy cells were tested with an incomplete drug library. In terms of low toxicity, mid- and lower ranking substances, like romidepsin and fludarabine phosphate, seem more promising. Itraconazole also showed low toxicity, but as noted above, it actually did not have an effect on cancer cells, either. Our calculations revealed that GI${}_{50}$-based toxicity ranking of substances is relatively similar to PoD-based assessment.

## 4. Discussion

In the present study, we investigated three commonly used measures of effect on a wide range of glioblastoma models, exposed to a large library of candidate drugs. We introduced a statistically founded definition of point-of-departure (PoD) and adapted the curve fitting procedure to produce physically plausible confidence bands. By choosing state-of-the-art technologies, we believe our data are a solid fundament for the development of our computational measures. We do not attempt to answer the question: “Which substance is the most suitable for treating a specific disease.” The reader should be aware that in silico experiments alone presently cannot give a conclusive answer to this question. Rather, our question is: “Which computational methods are suitable for identifying promising drug candidates in early stages of substance selection.” We use glioblastoma—a tumor type that is extremely hard to treat and presenting an urgent clinical need—as a model, but the computational methods are generic and applicable to determining substance toxicity on all kinds of cells. Our lab has established a quality control system in order to standardize our data generation and acquisition procedures, aiming to elevate the relevance of our research for clinical application [22]. To this end, we were inspired by the Guidelines On Target Assessment for Innovative Therapeutics (GOT-IT), which incorporate target-drug concentration range validation [28]. As far as determining the right dosage when translating from in vitro to human application is concerned, allometric scaling, pharmacokinetics/pharmacodynamics, and physiologically-based pharmacokinetic models can be used [29].

All three of the investigated measures of effect produced reasonable results on our 3D glioblastoma cell lines and the applied drug library. The results were not identical, but sufficiently similar. GI${}_{50}$ and AUC were highly correlated, leading to almost identical selection of substances. The correlation between GI${}_{50}$ and PoD was also high, but PoD sometimes led to a different selection. Substances that showed an effect at the beginning, but whose effect never reached 50%, were detected by PoD, but not by GI${}_{50}$. In addition, we observed that all criteria were somewhat susceptible to instabilities in the numerical fitting algorithms. We therefore advise computing both GI${}_{50}$ and PoD and, in the case of a large discrepancy (e.g., a non-effective drug by one criterion appearing among the top 20 according to the other), manually resolving it.

Despite producing similar results, one has to bear in mind that the three methods measure related, but different aspects of drug effect: GI${}_{50}$ relies on a point-estimate of the effect; PoD is derived from its confidence band; and AUC is the integral of the drug effect function. While one could—hypothetically speaking—construct some arbitration or balancing algorithm to combine the three results, such a method would lack a theoretical foundation. Another point worth noting is that the absolute values of the three measures are not directly comparable, which is why we needed to use the rankings. While GI${}_{50}$ and AUC estimate the true properties of the tested drug itself, PoD quantifies our confidence of having observed an effect in the concrete experimental setup. Using more data, e.g., by making multiple measurements, is bound to narrow down the confidence band and, consequently, lower the PoD.

Applying our calculation strategies on control cells, we benchmarked our selected high potency drugs for their adverse effect on non-cancer cells. Since many of the substance targeting molecular and cellular signaling pathways are not exclusively active in cancer cells, it is expected that drug candidates also affect our control counterparts. Looking at the concrete drug data of our study, we see that several top performers possess strong effects on non-cancer models. Those drugs shall be considered with caution for further consideration in follow-up studies. Mid-ranked therapy potency seem candidates rather attractive, such as romidepsin and fludarabine phosphate. In the end, any list of drug effects acquired on disease models only must be evaluated on an individual case basis. For that, the activation level of the dedicated drug target in cancer cells compared to the non-cancer area may help to predict susceptibility to the drugs and indicate off-target risk. This kind of research is actually a focus of our group and has been recently published in the context of the notch pathway inhibitor [30] or glutaminase 1 inhibitor [31]. However, the present study focuses on the computational method development for evaluating functional omics—interrogating the parameters of drug effect severity in cancer and non- cancer cells—to minimize the portfolio of different top substances for further investigation, rather than putting the biological context in focus. Similar to the cited papers, follow-up studies such as activation of the individual putative drug targets in relationship to the substance effect need to be performed in order to validate the suggestions.

The physical conducting of one-to-one comparative tests on healthy development in vitro models is favorable to predict toxicity, due to the power of real biologically acquired data. In this context, recently emerging synthetic cancer cell modeling technology, based on the introduction of cancer resembling genetic elements into healthy donor stem cells [32,33,34,35], offers a unique opportunity: given the step-wise introduction of multi-factorial alterations, one is able to model normal development, early stage malformation, and late stage disease progression in an isogenic, homogeneous single-cell of origin background. This setup may be more stable to score molecular associated, low adversity pharmacological perturbations as when relying on test matrices derived from different donors, as was the case in this study.

### Limitations

Our study focuses on the computational aspects rather than the clinical translational aspects of the retrieved drug effect results. We are aware of the following limitations in this study: (1) cell models—even the 3D stem cell centered approach, as in this project—insufficiently reflect tumor heterogeneity and tumor microenvironment, both known to significantly influence the chemotherapy resistance of the disease [36,37]; (2) any synergistic effect of the identified top drug candidates with radiation therapy or temozolomide, the standard of care treatment regime for glioblastoma [20], was not assessed; (3) we used a heuristic model for fitting the Hill curve to the observations; a theoretically founded error model would have been preferable; (4) as our study is based on data obtained from in vitro experiments, the results cannot be used for final drug selection. Instead, they should help the researcher to narrow down potential candidates for further in vivo and clinical studies.

## 5. Conclusions

We argue that relying on technological advancements in the field of disease modeling and the interrogation of transparent and accurate, preferably automated, laboratory workflow procedures are dominant influencers for determining relevant top substance candidates with high clinical relevance. In comparison to that, our data reveal that data post-processing strategies have lower potency to influence substance ranking, at least when using cell growth kinetics as phenotypic response readout. Given the plethora of existing service providers in the field of in vitro substance testing on living cells for the purposes of drug development or individualized medicine, our results may be relevant to a wider field of biotechnology than just cancer research. Due to our calculated discrepancies of top substance predictions in terms of their anti-cancer therapy potential and their off-target toxicology risk, we strongly advocate the interrogation of phenotypic drug response data acquired on proper non-disease control cell models in addition to cancer cell assays on order to deliver lab values with higher clinical applicability. However, confirmatory studies need to be conducted in order to validate this hypothesis. In addition, in vivo validation and combination therapy studies need to be conducted to validate the clinical potential of personalized anti-cancer treatments. However, in vitro screening approaches are the only realistic strategy to provide large-scale drug response analysis on living material within a rational time span and affordable budget requirement for wider application. Alternatively, tumor gene expression-based prediction of drug response emerges as a new avenue in personalized chemotherapy resistance for brain cancer patients [34,38,39] and shall be considered in the wake of AI technology developments. However, all computation-only-based measures remain in silico without real biologic/functional response measurement, sometimes leaving questions about their clinical relevance.

## Figures and Tables

**Table 1:** Top 20 drugs and their toxicities, ranked by GI${}_{50}$.

Substance	Rank (GI${}_{50}$)	Tox@GI${}_{50}$	Rank (PoD)	Tox@PoD	Rank (AUC)
Itraconazole	1	0.000	64	inf	10
Bortezomib	2	0.411	63	inf	1
Actinomycin D	3	0.596	1	0.636	6
Dinaciclib	4	0.586	2	0.641	8
Staurosporine	5	0.472	4	0.654	2
Ganetespib	6	0.566	3	0.600	3
Romidepsin	7	0.000	12	0.005	7
MLN9708	8	inf	5	inf	4
Carfilzomib	9	0.930	6	0.930	5
Homoharringtonine	10	0.659	7	0.696	9
PF-04691502	11	0.345	8	0.329	14
BAY80-6946	12	inf	9	inf	13
INK128	13	inf	28	inf	12
Obatoclax	14	0.534	10	0.434	11
Panobinostat	15	0.658	11	0.639	16
Auranofin	16	inf	13	inf	15
17-AAG	17	inf	14	inf	17
Idarubicin hydrochloride	18	0.834	16	0.790	19
Fludarabine phosphate	19	0.000	22	0.000	24
Daunorubicin hydrochloride	20	0.710	17	0.675	20

**Table 2:** Top 20 drugs and their toxicities, ranked by point-of-departure.

Substance	Rank (PoD)	Tox@PoD	Rank (GI${}_{50}$)	Tox@GI${}_{50}$	Rank (AUC)
Actinomycin D	1	0.636	3	0.596	6
Dinaciclib	2	0.641	4	0.586	8
Ganetespib	3	0.600	6	0.566	3
Staurosporine	4	0.654	5	0.472	2
MLN9708	5	inf	8	inf	4
Carfilzomib	6	0.930	9	0.930	5
Homoharringtonine	7	0.696	10	0.659	9
PF-04691502	8	0.329	11	0.345	14
BAY 80-6946	9	inf	12	inf	13
Obatoclax	10	0.434	14	0.534	11
Panobinostat	11	0.639	15	0.658	16
Romidepsin	12	0.005	7	0.000	7
Auranofin	13	inf	16	inf	15
17-AAG	14	inf	17	inf	17
Rigosertib sodium	15	0.477	24	0.477	25
Idarubicin hydrochloride	16	0.790	18	0.834	19
Daunorubicin hydrochloride	17	0.675	20	0.710	20
Vinflunine tartrate	18	0.404	97	inf	63
Doxorubicin hydrochloride	19	0.735	21	0.736	23
Ponatinib	20	0.854	23	0.894	21

## Abbreviations

The following abbreviations are used in this manuscript:

GI${}_{50}$	concentration required for 50% inhibition of growth
PoD	point-of-departure
AUC	area under the curve
IC${}_{50}$	inhibitory concentration 50%
CI	confidence interval
